# Quantification of hepatic steatosis in chronic liver disease using novel automated method of second harmonic generation and two-photon excited fluorescence

**DOI:** 10.1038/s41598-019-39783-1

**Published:** 2019-02-27

**Authors:** George Boon-Bee Goh, Wei Qiang Leow, Shen Liang, Wei Keat Wan, Tony Kiat Hon Lim, Chee Kiat Tan, Pik Eu Chang

**Affiliations:** 1 Department of Gastroenterology & Hepatology, Singapore General Hospital, Singapore, Singapore; 2 Department of Pathology, Singapore General Hospital, Singapore, Singapore; 3 Biostatistics Unit, Yong Loo Lin School of Medicine, National University of Singapore, Singapore, Singapore; 4 Duke-NUS Medical School, Singapore, Singapore

## Abstract

The presence of hepatic steatosis (HS) is an important histological feature in a variety of liver disease. It is critical to assess HS accurately, particularly where it plays an integral part in defining the disease. Conventional methods of quantifying HS remain semi-quantitative, with potential limitations in precision, accuracy and subjectivity. Second Harmonic Generation (SHG) microscopy is a novel technology using multiphoton imaging techniques with applicability in histological tissue assessment. Using an automated algorithm based on signature SHG parameters, we explored the utility and application of SHG for the diagnosis and quantification of HS. SHG microscopy analysis using GENESIS (HistoIndex, Singapore) was applied on 86 archived liver biopsy samples. Reliability was correlated with 3 liver histopathologists. Data analysis was performed using SPSS. There was minimal inter-observer variability between the 3 liver histopathologists, with an intraclass correlation of 0.92 (95% CI 0.89–0.95; p < 0.001). Good correlation was observed between the histopathologists and automated SHG microscopy assessment of HS with Pearson correlation of 0.93: p < 0.001. SHG microscopy provides a valuable tool for objective, more precise measure of HS using an automated approach. Our study reflects proof of concept evidence for potential future refinement to current conventional histological assessment.

## Introduction

Hepatic steatosis (HS) is defined by the abnormal accumulation of fat in liver hepatocytes and is an integral histological feature often evaluated in liver histological assessment. It can present in a variety of liver disease or injury with clinical implications dictated by degree of HS^1^. This take on additional significance in the context where there has been rising prevalence of liver diseases where HS may feature. For one, HS defines Non-Alcoholic Fatty liver Disease (NAFLD), currently the most common chronic liver disease worldwide, with global prevalence estimated at 25% of the world’s population^2^. Characterised by the excessive accumulation of fat (>5%) within the liver that is not attributable to alcohol, NAFLD epitomises a spectrum of disease ranging from simple steatosis, non-alcoholic steatohepatitis (NASH) to NAFLD related advanced fibrosis, cirrhosis or hepatocellular carcinoma (HCC)^3^. Considering the increasing rates of NAFLD related cirrhosis, HCC and indication for liver transplantation, NAFLD represents a substantial clinical and economic burden^4^. Similarly, HS can be also a feature in other liver diseases such as chronic viral hepatitis C, alcoholic liver disease or drug induced liver injury^5^. In addition, HS may co-exist with other chronic liver disease where it may impact on the natural history of the disease^6,7^.

Current histologic assessment of HS is performed semi-quantitatively, leaving the potential for intra- and inter-observer variability^8^. Furthermore, the semi-quantitative categorical scoring assessments may limit precision, especially in the context of subtle changes. Hence, a continuous reproducible quantitative scale may be better at characterising HS, particularly interval changes or therapeutic responses.

Second harmonic generation (SHG) microscopy is a novel optical imaging modality that can potentially identify and assess clinically pertinent aspects of liver histology^9^. Besides reliable staging of metavir fibrosis in rat models and human biopsy specimens in context of chronic hepatitis B (CHB)^10^, SHG microscopy has also used to analyze fibrosis in NAFLD cohorts. Our group developed the B-index, an algorithm based on several signature SHG parameters, to predict fibrosis in NAFLD^11^. This B-index provided excellent area under receiver operating characteristics (AUROCs) >0.90 to predict the various Brunt fibrosis stages in NAFLD. Separately, we developed an automated algorithm based on signature SHG parameters that reflect hepatic steatosis (HS) on liver histology. In this study, we explored the utility and application of SHG for the diagnosis and quantification of HS.

## Results

The baseline clinical characteristics of the study cohort are summarized in Table 1. The study comprised of 58.1% males of predominant Chinese ethnicity (84.9%), with a mean age of 51.5 ± 10.45 years (range 23 to 69 years). There were 43 NAFLD (50%), 23 CHB (26.7%) and 20 CHB-NAFLD (23.3%) subjects in the study cohort. Mean BMI was 29.7 ± 6.7 kg/m^2^ (range 17.6–50.5). Distribution of steatosis was reported as follows – no significant steatosis (18.6%), mild steatosis (38.4%), moderate steatosis (34.9%) and severe steatosis (8.1%). Of the 43 patients with pure NAFLD, 81.4% had NASH (based on overall pathologist’s intepretation) and 34.9% had advanced (stage 3 or 4) fibrosis. Of the 20 patients with concomitant CHB-NAFLD, 40% had NASH and only 1 patient (5%) had advanced fibrosis. Median liver stiffness measurement evaluated by transient elastography was 11.0 kPa (range 4.1 to 58.1 kPa).

**Table 1:** Baseline characteristics of NAFLD cohort.

	All subjects (n = 86)
Mean age (years)	51.5 ± 10.5
Male gender	50 (58.1%)
Chinese ethnicity	73 (84.9%)
BMI (kg/m^2^)	29.7 ± 6.7
Median LSM (kPa)	11.0 (4.1–58.1)
Albumin (g/L)	38.5 ± 5.3
Bilirubin (µmol/L)	16.5 ± 12.8
Alanine transaminase (U/L)	75.8 ± 47.8
Aspartate transaminase (U/L)	55.9 ± 30.4
Platelet count (x10^9^/L)	215 ± 75
Prothrombin Time (s)	10.4 ± 0.7
Creatinine (mmol/L)	80.7 ± 22.8
Histological steatosis grade	
No steatosis, S0 (<5% steatosis)	16 (18.6%)
Mild steatosis, S1 (5–33% steatosis)	33 (38.4%)
Moderate steatosis, S2 (>33%-66% steatosis)	30 (34.9%)
Severe steatosis, S3 (>66% steatosis)	7 (8.1%)

Data are presented as mean+/− standard deviation, number and percentages and median + range.

NAFLD: non-alcoholic fatty liver disease, BMI: Body Mass Index, LSM: Liver Stiffness Measure.

The three expert liver histopathologists reviewed each histological specimen and independently scored the severity of steatosis, providing a steatosis percentage for each specimen. The inter-rater agreement of percentage steatosis between the 3 pathologists was high with an intraclass correlation of 0.92 (95% confidence interval 0.89–0.95, p < 0.001). Severity of steatosis degeneration (SSD) was independently evaluated in an automated fashion using the SHG algorithm, which also provided an automated steatosis percentage for each unstained specimen. There was an excellent correlation (Spearman correlation 0.94, p < 0.001) between the histopathologists’ assessment of percentage steatosis and the SSD calculated by the SHG method.

**Figure 1:**
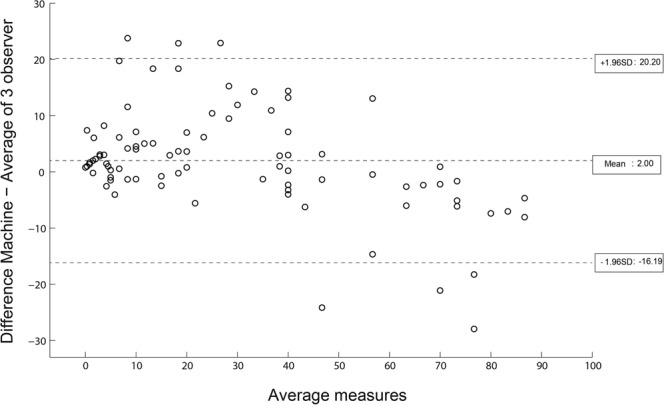
Bland-Altman plot comparing steatosis assessment using SHG microscopy vs. histopathologist assessment. Illustrates the initial Bland-Altman plot comparing steatosis assessment using SHG microscopy with standard histopathologist; mean difference of 2% with a 95% confidence. interval (±1.96 standard deviation) of −16.19 to 20.20% was seen.

The initial Bland-Altman plot of the entire cohort (n = 86) demonstrated a mean difference between pathologists’ and SHG assessment of steatosis of 2% with a 95% confidence interval (±1.96 SD) ranging from −16.19% to +20.20% (Fig. 1). Firstly, we examined if there was any potential difference in accuracy of SHG assessment of SSD between our five-by-five sampling method (n = 48) versus full imaging (n = 38) of the liver tissue sample. Pearson correlation between the two methods was almost equal, r = 0.982, with a small mean difference of −0.60 ± 8.48%. Hence there was no significant difference in the SSD result when assessed using the five-by-five sampling method compared to imaging of the full tissue sample.

**Figure 2:**
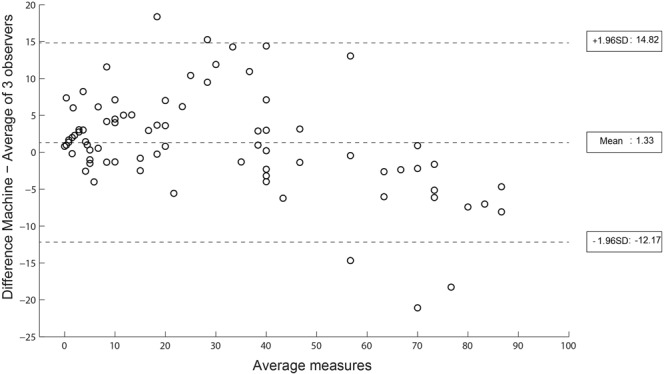
Bland-Altman plot comparing steatosis assessment using revised SHG microscopy algorithm vs. histopathologist assessment. Illustrates the improved revised Bland-Altman plot, correcting for systemic factors of discrepancies, where the mean difference between SHG and pathologist assessment of steatosis was reduced to 1.33% with a 95% confidence interval (±1.96 standard deviation) of −12.17 to 14.82%.

Subsequently, we interrogated the subjects with unexpected interobserver discrepancy to investigate if there was a systematic reason for the poor correlation between SHG’s assessment of SSD to the histopathologists’ assessment of steatosis. We found three broad factors that contributed to the discrepancy in results. Firstly, a subset of cirrhotic patients had fragmented liver biopsies. Such tissue fragmentation resulted in an inaccurately low SSD as the algorithm overestimates the area included as the denominator for calculation of steatosis. Secondly, a few biopsies showed cytoplasmic rarefaction of hepatocytes that resulted in the automated SHG microscopy algorithm misidentifying these areas as areas of steatosis, thus providing an inaccurately high SSD. This histological artefact is a result of suboptimal tissue fixation and/or preservation. Thirdly we also recognise that in biopsies that suffer from other technical issues such as suboptimal thickness (less than 5 um) or presence of non-representative tissue such as blood clots, these factors can also affect the algorithm performance. This led us to refine the SHG algorithm by excluding these outliers manually. The revised Bland-Altman plot (Fig. 2) after revision of the algorithm demonstrated a mean difference between SHG and pathologists’ assessment of steatosis of 1.33% with a 95% confidence interval (±1.96 SD) ranging from −12.17% to +14.82%.

## Discussion

Our study demonstrates the ability of our novel algorithm to accurately quantify HS in an automated fashion using SHG. There was good correlation with traditional microscopic examination of H&E stained slides by corresponding histopathologists. More importantly, this algorithm intuitively represents a more reproducible and precise measure of HS compared to the current semi-quantitative method of assessment.

The degree of abnormal amount of HS is an important clinical issue in the context of characterising liver diseases. Previous studies have reported inconsistent variability in estimation of liver fat percentage. Hall and colleagues suggested that while experienced pathologists had good agreement in estimating HS and assigning steatosis grade, compared to more precise digital image analysis measurement of fat, pathologists generally overestimates HS^12^. This was also echoed by similar findings from other studies^13–16^. Separately, El-Badry *et al*. showed poor agreement among a group of expert pathologists with regard to grading liver steatosis, with an interclass correlation of 0.57^17^. Other studies have shown variable strength of concordance^8,18^, demonstrating the wide range of subjectivity and possible issues with reproducibility. In addition, comparisons between studies may also be difficult to interpret in this context. In this respect, several experts have advocated for a standardised way of quantifying HS, particularly as a continuous variable^19^.

Our algorithm is the first study that utilises SHG microscopy technology for automated grading of HS. SHG microscopy is a novel optical method increasingly used in characterising histological tissue such as neurons and collagen by analysing spatial architectural features and signals produced^20^. This has previously been described in detail and has been shown to be reliable in assessing liver fibrosis in chronic viral hepatitis and NAFLD^10,21,22^. With increasing interest in the SHG microscopy, our current study demonstrates the utility and potential of SHG microscopy for expanded indication, such as HS and possibly other pertinent histological features.

There are several advantages of our algorithm. Firstly, it is comparable to traditional histologic assessment by experienced liver histopathologists. Secondly, by expression in a continuous scale, the quantification of HS can be more precise and allow for detection of subtle interval nuances that may not be so apparent using traditional approach. This takes on more relevance in the context of the many current clinical trials interrogating the effectiveness of therapeutic agents for NAFLD^23^. Thirdly, using this system minimises the operator dependent subjectively compared to traditional assessments, allowing for more standardization of measurement and reproducibility. In addition, by assisting in the automated quantification of HS, the pathologist is unburdened to review other pertinent histological features. This ancillary tool complements and supports the pathologist for in-depth examination of the tissue specimen, while potentially improving throughput and efficiency. Separately, sample preparation for assessment is fairly straightforward, SHG microscopy can be performed using either frozen fixed or paraffin embedded tissue. Furthermore, in contrast to some other techniques in identifying HS via special staining, no specific staining steps need to be undertaken in SHG microscopy, making the process fast and easy to execute. We acknowledge that there are other label free imaging techniques for the investigation of hepatic steatosis, such as Coherent Anti-Stokes Raman Scattering (CARS) or Stimulated Raman Scattering (SRS) microscopy^24,25^. Some studies utilize a combination of SHG and CARS imaging to quantitatively assess fibrosis and steatosis respectively^26^. Our study utilizes a single platform, SHG to perform both functions of assessing fibrosis and steatosis. One additional advantage of our SHG platform is that we can utilise paraffin embedded sections, which is used in most standard practice of real world clinical workflows, while fresh or frozen specimens are usually needed in CARS and SRS microscopy.

There remain several limitations of our study, including the cross sectional nature of the study and modest sample size from a single centre. Future studies involving larger cohorts from multiple centres which are currently being planned, are required to validate our algorithm.

In our study, we have also seen the impact of tissue quality on the algorithm performance. Prior to scanning the tissue for analysis, we recommend that a quality control step to ensure optimally fixed and prepared representative liver tissue specimen be used.

Along similar lines, future exploration of quantifying other pertinent histological features such as balloon degeneration and lobular inflammation using SHG would also be impactful in providing more comprehensive analysis, particularly in the setting of NAFLD.

In conclusion, SHG microscopy provides a valuable tool for an objective, more precise measure of HS using an automated approach. Our study reflects proof of concept evidence for potential future refinement to the current conventional histological assessment.

## Materials and Methods

### Study population

In this retrospective study, suitable subjects with a clinical diagnosis of CHB and/or NAFLD who underwent liver biopsy for clinical indications between 2006 and 2016 in the Department of Gastroenterology and Hepatology of the Singapore General Hospital were identified from the department registry database. CHB subjects were included if they were positive for hepatitis B surface antigen (HBsAg), regardless of hepatitis B e antigen status. All CHB patients were treatment-naïve. The clinical indication for liver biopsy in CHB patients was to evaluate severity of fibrosis for consideration of antiviral therapy. NAFLD subjects were included if they fulfilled the definition of NAFLD with >5% hepatic steatosis on liver biopsy in the absence of viral hepatitis, significant alcohol consumption (defined as >21 units of alcohol per week in males and >14 units in females), autoimmune and hereditary liver diseases. In NAFLD subjects, liver biopsy was performed to evaluate the severity of steatosis, inflammation and fibrosis for diagnostic and/or prognostic indications. The CHB-NAFLD group comprised of subjects with clinical evidence of both conditions. These patients were all HBsAg-positive with evidence of steatosis on liver ultrasound and >5% steatosis on liver biopsy in the absence of significant alcohol consumption. The indication for liver biopsy in this cohort was either to evaluate the predominant etiology contributing to active hepatitis or assessment of fibrosis severity for prognostic indications. Archived, formalin-fixed, paraffin-embedded liver biopsy samples from 86 subjects who fulfilled the above criteria were retrieved for analysis. The study protocol was approved by the SingHealth Centralised Institutional Review Board E, including waiver of need to obtain informed consent. All research was performed in accordance with Singapore guidelines for good clinical practice.

### Histological assessment

The liver biopsy samples were processed as per routine clinical practice. 5 µm-thick sections were cut from archived liver biopsy tissue and the anonymised specimens were sent for SHG imaging. The same sections were subsequently stained with hematoxylin and eosin (H&E) stain for the histopathologists’ blinded assessment of HS. Biopsy samples were reviewed in a blinded fashion by a panel of 3 experienced liver histopathologists who independently scored the severity of hepatic steatosis using the Brunt classification (S0: <5% steatosis, S1: 5–33% steatosis, S2: 33–66% steatosis, S3: >66% steatosis)^27^.

### Image acquisition

Unstained biopsy tissues of the 86 samples were imaged by the commercially available Genesis system (HistoIndex Pte. Lte., Singapore), in which second harmonic generation (SHG) microscopy was used to visualize collagen and two-photon excited fluorescence (TPEF) microscopy was utilized for visualization of the pertinent cell structures. The settings of the laser were previously described^10^. In brief, the setup involves a laser passed through a pulse compressor (for group velocity dispersion) and an acoustic-optic modulator (for power attenuation), following which, is routed by a dichroic mirror, through an objective lens to the tissue sample, where TPEF emission and SHG signal are collected and processed for detection. The Genesis system is a closed system, where only the objective lens is adjustable (10x, 20x, and 40x), while all other components like condenser, dichroic mirror, collection filters, and collection frame rate are not adjustable^20^.

The samples were laser-excited at 780 nm, SHG signals were recorded at 390 nm, and TPEF signals were recorded at 550 nm. The optical filter bandwidth was 11 nm and 88 nm for SHG and TPEF respectively. Images were acquired at 20x fold magnification with 512 pixel × 512 pixel resolution, and each image tile had a dimension of 200 μm × 200 μm. To cover most of the tissue areas, 10 five-by-five multi-tile images were randomly acquired for the 86 samples with a final sampling size of 10 mm^2^. Furthermore, 38 out of those 86 samples were fully imaged to analyse the sampling error associated with the five-by-five acquiring.

### Quantification algorithm of steatosis degeneration

We recognise that steatosis is the accumulation of abnormal amounts of lipid within hepatocytes. During the tissue processing, the lipids are leeched out and is seen as cytoplasmic vacuoles / holes on histology. To differentiate a steatotic hole from other non-steatotic holes (such as bile duct lumens, vascular spaces, sinusoidal spaces and artefactual clefts), we used additional subtle histological features; The steatotic vacuole is intracellular and usually pushes the hepatocyte nuclei to one side. Although macrovesicular steatosis is the predominant type of steatosis seen in NAFLD, there can be accompanying intermediate-to-small droplet steatosis, which can be identified histologically as smaller lipid vacuoles within the hepatocytes. In early and milder forms of steatosis affecting adults, the lipids usually accumulate around the zone 3 hepatocytes. All these features were taken into consideration to assist us in the algorithm development for the histological ascertainment of true lipid steatosis^28^. Accordingly, the possible non steatotic holes, such as vascular/bile duct lumens or areas prone to tissue artefacts that may create non-steatotic holes, such as the edges of the biopsy where there is more tissue fragmentation, were minimised in the computation of steatosis quantification.

**Figure 3:**
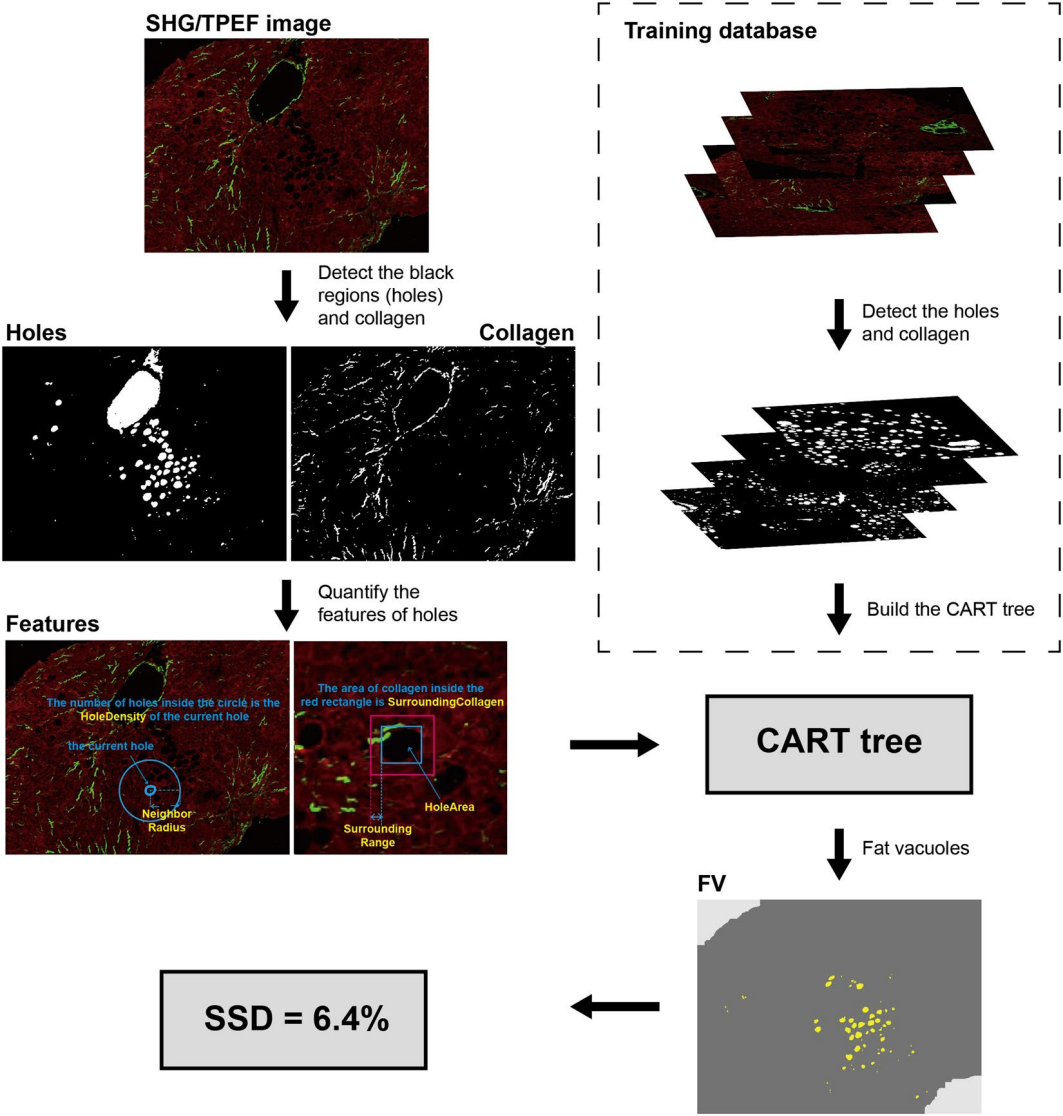
Flowchart of the quantification of steatosis degeneration. Illustrates the flowchart of the quantification of steatosis degeneration. All the holes in the input SHG/TPEF image were detected and classified by the CART tree, which was built based on the training database. The fat vacuoles (FV) were detected and used to calculate the severity of steatosis degeneration (SSD).

Following are details about the major steps of the processing flow (Fig. 3). Firstly, all the empty holes inside tissues were detected from the TPEF channel by the Ostu’s automatic threshold method^29^. Each hole with area smaller than a predefined threshold was considered as a noise to be ignored. The threshold was defined by the minimum area of established fat vacuoles.

Secondly, fat vacuoles were distinguished from others using a decision tree, which was constructed by classification and regression tree (CART) method^30^.

The CART model was built based on the training image database. All the holes including fat vacuoles, vessels and cracks are detected. Then, the fat vacuoles were identified from all holes by an expert pathologist. The features used in the decision tree were decided by the holes and their surrounding collagen, such as distribution density of hole, width and length of hole, hole solidity, the area of surrounding collagen of hole, etc. The threshold values of the parameters to identify fat vacuole were determined by the CART method automatically.

**Figure 4:**
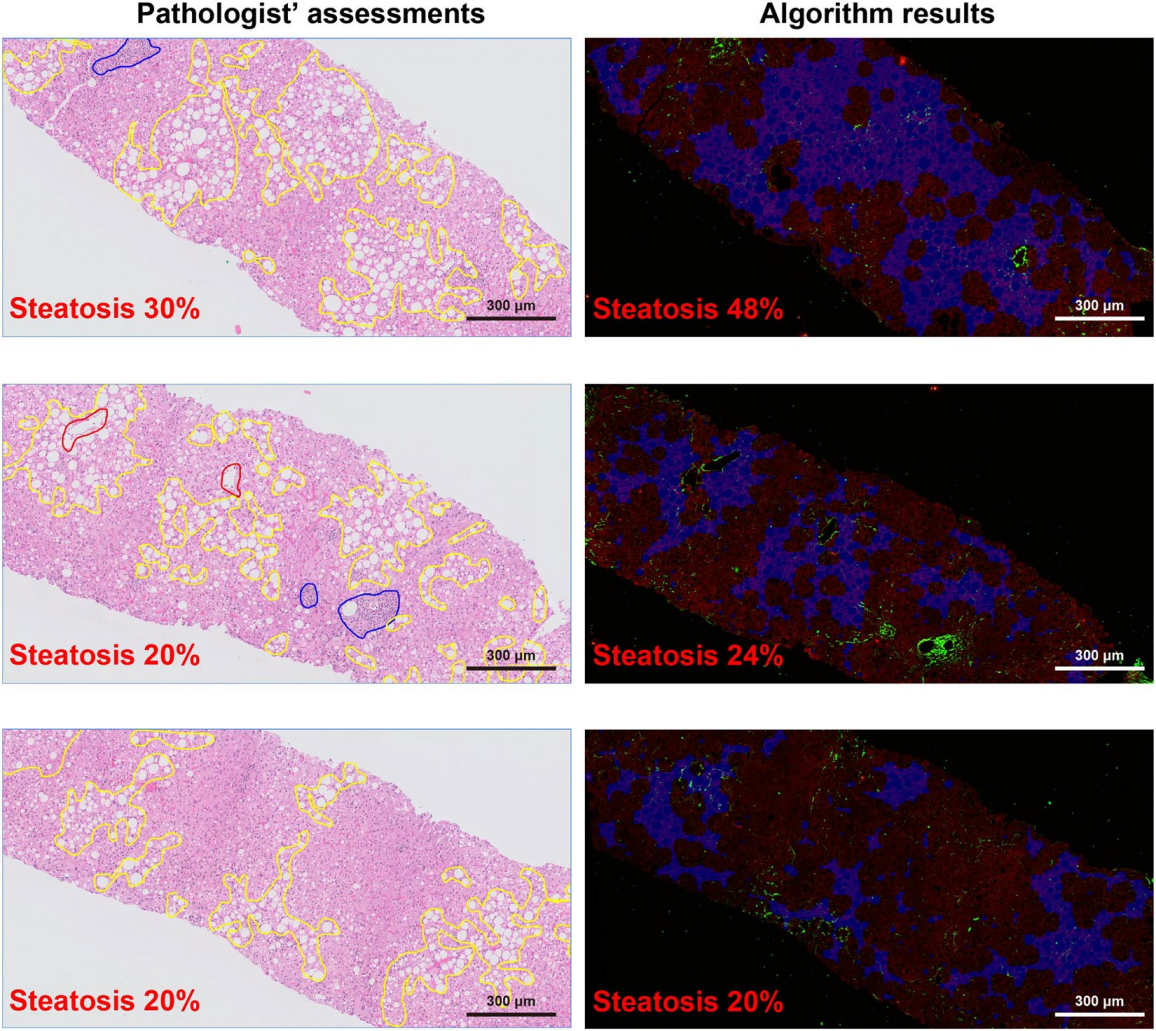
A comparison between pathologists’ assessments and the SHG Algorithm SSD. Illustrates examples of pathologist assessing hepatic steatosis using standard hematoxylin & eosin stained slides on the left and corresponding SHG algorithm derived assessment on the right. The regions with steatosis degeneration in H&E images were circled with yellow curves, and the severity of steatosis was assessed by pathologist. The portal tracts and central veins were circled with blue and red curves, respectively. In the SHG/TPEF images, the regions with steatosis degeneration were automatically labelled in blue and quantified by the algorithm. There was good correlation between the two methods of assessment.

Finally, the severity of steatosis degeneration (SSD) was estimated by the percentage of steatosis regions in the entire biopsy tissue and reflects the percentage of cells with fat vacuole, as identified by the algorithm. The entire biopsy tissue was detected from the TPEF channel automatically. The steatosis regions consist of not only the fat vacuoles with high densities, but also the hepatocytes around the vacuoles. Fat vacuole candidates were ignored if there was no other hole around. The SSD ranges from 0%–100%, with 0% reflecting absent steatosis degeneration while 100% representing the most severe of steatosis degeneration. Figure 4 illustrates the comparison between pathologist and SHG algorithm SSD assessment of selected specimens.

### Statistical analysis

Correlation between individual pairs of pathologists’ assessment of steatosis and correlation between the mean of pathologists’ assessment of steatosis and the SSD were calculated using Spearman correlation. Inter-rater agreement between the 3 pathologists’ assessments of steatosis and inter-rater agreement between Pathologists’ assessment of steatosis and the SSD were assessed by intra-class co-efficient (ICC). The difference between Pathologists’ assessment of steatosis and SSD was demonstrated by the Bland-Altman plot. Statistical analysis was done with SPSS ver 23 (Chicago, IL, USA). Statistical significance level was set at p < 0.05.
